# Motivations of South African physicians specialising in public health

**DOI:** 10.1080/16549716.2018.1475039

**Published:** 2018-05-29

**Authors:** Virginia Elizabeth Melvill Zweigenthal, William M. Pick, Leslie London

**Affiliations:** School of Public Health and Family Medicine, University of Cape Town, Cape Town, South Africa

**Keywords:** Community medicine, education, medical, graduate, human resources, public health, South Africa

## Abstract

**Background**: South African physicians can specialise in public health through a four-year ‘registrar’ programme. Despite national health policies that seemingly value public health (PH) approaches, the Public Health Medicine (PHM) speciality is largely invisible in the health services. Nevertheless, many physicians enrol for specialist training.

**Objectives**: This study investigated physicians’ motivations for specialising in PHM, their intended career paths, perceptions of training, and perspectives about the future of the speciality.

**Methods**: Focus groups and in-depth interviews were conducted with specialists-in-training and newly qualified specialists, and thematic analysis of transcripts was performed.

**Results**: Motivations, often driven by difficult experiences as young physicians in poorly resourced clinical settings, stemmed from a commitment to improving communities’ health and desire to impact on perceived failing health systems. Rather than ‘exiting’ the South African health service, selecting PHM specialist training enacted participants’ ‘loyalty’ to population health. Participants anticipated carving out their own careers due to an absence of public sector career paths. They believed specialists’ contribution centred on providing ‘public health intelligence’ – finding and interpreting information; supporting services through management and leadership; and inputting into policymaking and planning.

**Conclusions**: Competencies of PHM specialists should be refined to inform and improve management of this scarce human resource for health. This is particularly important given the proposed major health reforms towards universal health coverage in South Africa presently. In addition, findings highlight the importance of physicians’ early work experiences where avenues for expressing ‘voice’, mediated by ‘loyalty’, could be utilised to improve public sector health systems.

## Background

Since apartheid, South Africa’s (SA’s) health system has been extensively restructured, creating 52 districts within 9 provinces. Further restructuring is proposed in the 2017 National Health Insurance (NHI) policy paper [1] which details plans for an equitable health system towards universal health coverage. Although reform is underpinned by public health (PH) concerns, policy has focused on building clinical services.

SA trains PH personnel through postgraduate public university degrees, typically the Master of Public Health (MPH), which admit graduate health and social science students, including physicians, but curricula are not standardised. There is also funding for salaried physician specialists-in-training – ‘registrars’ in Public Health Medicine (PHM). Despite SA producing many skilled PH practitioners [2], national human resource policy is silent about a PH workforce to monitor progress, identify service priorities and implement strategies [3]. Until recently, there were few PHM posts outside university academic positions, and postgraduate PH qualifications are rarely required for appointments in the state health sector.

PHM, a small speciality in SA, was established in 1984. In March 2018 there were 150 registered specialists with the Health Professions Council of South Africa (HPCSA). Its current scope of practice is not with direct patient care. Similar to other countries, work is at local, regional and national levels [4–7], often in policy and leadership positions [8,9], in public institutions, in academia and research [5,10]. Specialists qualify through four-year university-based training progammes accredited by the HPCSA. Universities award a research-based Master of Medicine (MMed) towards this specialisation. Since 2011, specialist registration requires physicians to complete both the MMed and a fellowship from the College of Public Health Medicine (CPHM), a national body of peers that oversees exit exams for the speciality.

Similar to North America, the UK, France and Italy [11], training in SA is based on an apprenticeship model, where registrars work full-time in service settings. As found elsewhere [11], attachments and theoretical learning for SA trainees are heterogeneous, but college exams have standardised outcome competencies and curriculum descriptions.

This study was conducted in 2012 to understand factors attracting physicians to PHM training considering their ill-defined career paths; inform discussions to revise SA’s PHM curriculum; and contribute to national health workforce policy discussions. It contributes to current discussions about the recognition and definition of a PH medical speciality internationally [11]. The study explored the motivations of registrars who chose four-year PHM training rather than the MPH degree; the impact of prior work experience and medical training on their choice; their anticipated career paths; and reflections about potential roles of PHM specialists in SA.

## Methods

### Study setting

Research was conducted among registrars enrolled in three of the seven SA institutions based in two urban centres offering specialist training. These centres, over 1500 km apart, host the two largest PHM training programmes. The third institution is located in the same centre as one institution.

### Overall research design

Focus group discussions (FGDs) and in-depth interviews (IDIs) were ideal for exploring the complex phenomena in this study: motivations, intentions, opinions and perceptions. Registrars were at similar points in their careers and FGDs enabled discussion about common experiences. IDIs were conducted with recently exiting specialists. As they were employed full-time, focus groups were not possible. IDIs enabled reflection on training programmes and specialists’ possible roles.

### Sampling of informants

All registrars based in these centres were invited to participate in the study. Seventeen of the 19 registrars participated, over half the 32 trainees nationally. One from each centre did not participate. In addition, three of the four specialists who exited the training programme from the institution where researchers were based participated. One, based in another centre, did not participate as no suitable time for the interview could be found.

### Data collection

Three FGDs focus groups with registrars were held. Two mixed registrars from institutions based in one centre, while the third was with registrars from the second centre. In-depth interviews were used to collect data from the recently qualified specialists at a convenient time and venue for participants. Participation was voluntary and all signed consent and agreed for interviews to be digitally record. Participants could withdraw and were assured of their own and their institutions’ anonymity. Interviews, in English, were transcribed verbatim by a contracted professional, and stored in password-protected files.

### Analytical approach

The researcher (VEMZ) immersed herself in the data, listening to recordings, reading transcripts and corrected errors. The transcribed data was analysed, using Atlas ti 7.0.92; and using Bowling’s approach, key themes were first identified and then clustered into dominant themes [12]. To assure rigour, an information-rich transcript was analysed by an external experienced qualitative researcher, who confirmed the emerging themes. After all transcripts were analysed, a narrative of the findings was written. Transcripts were reviewed and quotations best voicing themes were inserted into the narrative. These are FGD 1–3 for FGDs, and IDI 1–3 for IDIs. Reflexivity contributes to research validity [13], and an author (VEMZ), a PHM specialist, conducting the interviews, was mindful of perhaps being an authority figure for participants.

Initially the ‘push–pull’ framework was used to analyse the emerging dynamic related to registrars’ decisions to specialise in PHM. This framework is commonly applied to explain health worker migration and career decision-making [14]. However, it did not explain informants’ underlying motivations to specialise. We were alerted to an alternative framework developed by Hirschman of *Exit, Voice and Loyalty* [15] which more satisfactorily explained the dynamic underlying informants’ career decision-making and motivations. This model incorporates individuals’ motivations for choices made when confronted with difficult work contexts – ‘go or stay’. It considers individuals’ underlying motivations/vocation, their ‘loyalty’ that mediates decision-making to ‘exit’ or remain, and ‘voice’ agency [15]. Whilst highlighting underlying motivations, this model does not fully explain all the factors involved in participants’ decision-making.

The study was approved by the Human Research Ethics Committee at the University of Cape Town (Ref No: 251/2010).

## Results

Registrars came from a wide range of training and service backgrounds. They graduated from seven of the eight SA universities producing physicians. Five trained at an institution whose registrars were not sampled, and three graduated from each of the three institutions sampled. Their median work experience was seven years (IQR: 6–9.5) prior to the FGDs. The gender distribution (5 males and 12 females) was similar to the registrar population over the past decade.

The three recently qualifying specialists trained as physicians at three different universities, and had between three and eight years work experience prior to registrar training. All were women, reflecting the national profile, as all eight specialists exiting training over that period were women. Two worked for different non-governmental organisations (NGOs) supporting national health system reform and one worked in academia.

Bar two, all participants had recently worked clinically as junior physicians in SA urban, peri-urban and rural district hospitals. Of the 20 interviewed, 15 (75%) were women.

The following themes are presented: for registrars leaving clinical work, themes are their *negative experiences* as junior doctors, their perception of the *limited nature* of clinical work and *personal preferences*. Rather than leaving the health sector, they were committed to improving the health status of people and chose to study and work in PH to express this. Themes related to their selection of PHM are their value of *becoming specialists*, and their perception of the *superiority* of their theoretical and experiential training compared to master’s courses. Themes related to the PHM speciality are its *broad scope* with *competencies overlapping* with other PH professionals. This was related to PHM having an uncertain identity and career paths. This links to themes about their medical school experience of PH teaching which was *uninspiring* and *unrelated* to clinical work, and the *invisibility* of PHM. Regarding the speciality’s future, in view of health care reform underway in South Africa, themes are their *optimism* for PHM and their own future, yet *uncertainty* anticipating competition with other PH professionals

### ‘Exit’ – leaving clinical work

#### Negative experiences

Overwhelmingly, informants’ clinical experience as junior physicians was frustrating. Some found working solo in busy casualty wards stressful, managing patients with little supervision. Working unsupported, especially at night, or being expected to deliver care with sub-optimal resources was an impetus to leave clinical medicine:

You’re just there to be … the hero who saved the day without the support from your superiors and the system as a whole. Sometimes you’d improvise and do the wrong thing. [If] all goes well … you’re happy, but if that one time … it doesn’t go well, someone will come. I wouldn’t want to live like that all my life, ducking and diving. (FGD1)

#### Limited nature

Another theme from all FGDs was disillusionment with clinical medicine, due to seeing patients repeatedly for similar preventable conditions – the ‘revolving-door’ phenomenon, as well as a sense that clinical medicine had limited impact:

I … realized … that what you do as a doctor with an individual patient doesn’t make as much difference as what’s done higher up the systems of health care delivery. And so I was quite disillusioned with medicine at that point (FGD2).

#### Personal

Registrars raised personal reasons for leaving clinical work such as night calls, the need for personal growth, and ‘burn out’:

‘After having done internship and those crazy hours you start feeling normal again … You’re still a doctor, you’re still making some difference but you’re not killing yourself in the process’ (FGD3).

However, exiting clinical work was also voiced as a loss and registrars missed being at the frontline of health care delivery:

‘ I used to be able to help everyone … but now I can’t … Before I started in public health, it was a big argument in my mind, am I going to give up being a doctor?’ (FGD1).

### ‘Loyalty’

Their frustration with clinical work led them to want to change the health services rather than leave medicine or emigrate. This triggered a search for training that would have impact. Those who enjoyed clinical work also wanted to work at a systems level. These physicians appreciated that PH practice values other health professionals, working collegially and as equals, and this drew them to PH:

It was all along the lines of wanting to make a difference (FGD2).

My mind started running wild with ideas on how things could change (FGD3).

A doctor is only a small part of the delivery of health care and everybody else is equally important. So that’s a very big thing that comes out in public health (FGD2).

### ‘Voice’ – specialising in PHM

PHM’s scope was the dominant theme motivating participants – impacting on health delivery, preventing ill-health and addressing determinants of health in communities. Registrar training provided the necessary broad skills and opportunities:

‘Thinking about the population and about risk factors … evaluating health systems, health exposures, health risks, health outcomes. That’s what ultimately led me to … public health’ (FGD2).

Informants desired to move into policy or decision-making positions to transform ineffective services they experienced. They wanted to manage ill health comprehensively, and focus on determinants of diseases. They gave up their clinical career plans.

PH’s altruistic nature and commitment to equity attracted informants. One commented on PH’s concern for ethics:

‘There’s little dignity in going from one place to the other, long waiting times… There’s always this golden thread of, “What does it mean for the individual, the population, and does it restore dignity?”’ (FGD2).

**Figure 1.**
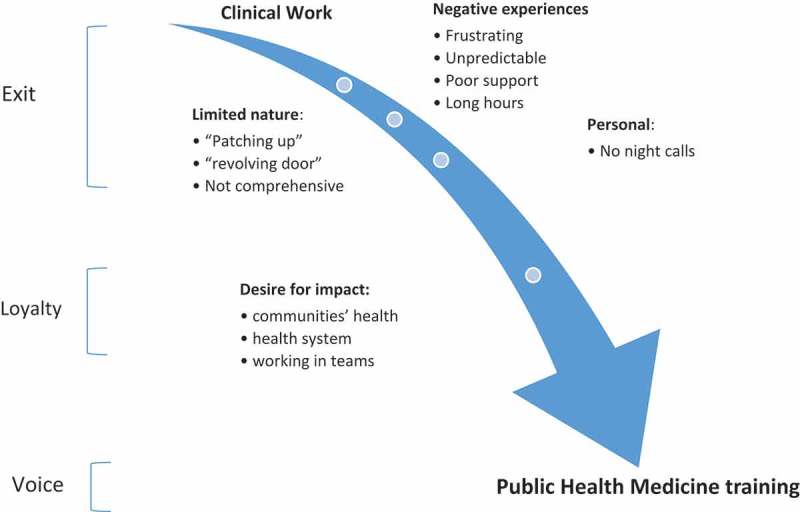
Motivating factors for PHM training.

Factors contributing to registrars exiting clinical work, their loyalty to people’s health resulting in their decision to undergo PH training, are depicted in Figure 1.

### PHM’s breadth – a double-edged sword

A prominent theme was PHM’s broad disciplinary domain, and competencies which are shared with and overlap with other PH professionals. Participants from all FGDs were attracted to PHM’s quantitative orientation anticipating work that included ‘measurement’.

A minor theme was that the breadth of PHM was attractive, as it encouraged thinking beyond immediate health problems and promoted problem-solving. One registrar characterised PHM as a ‘speciality for non-specialists’:

In clinical medicine … you’re going into the exact route that someone else has followed before you. You’re actually learning more of less. Some want to know a bit about everything and try to bring things together … Public health is great for people who think like that (FGD1).

However, overwhelmingly informants believed PHM had an uncertain identity and poor career profile, which they speculated was related to overlapping domains with other PH professionals. Participants were critical of the lack of advocacy for the speciality and there was extensive discussion about this:

‘I remember going to some consultants and asking, “So what actually is [*public/*]community health?” And I could not get a consistent answer’ (FGD3).

### Public health learning as medical students

Overwhelmingly, participants reported that prior PH learning played little role in their decisions to specialise. Courses were seen as being ‘completely boring and irrelevant’, inferior to clinical work. PH subject matter was often confused with Family Medicine. There was some exposure to community settings, highlighting social determinants of disease:

‘It’s understanding where people get their drinking water … where are children playing, who is looking after them, what are they exposed to?’ (IDI 2).

Overwhelmingly, participants saw PH exposure as too rushed or dense with many assignments and little time to reflect:

‘It was doing a project, getting it over, swot learning … not understanding what you’re doing’ (IDI 1).

A pervasive theme was that PH was invisible in medical training, and there were few PHM role models for students intending to work at a population level. Informants only became aware that PHM was a medical speciality when they enquired about MPH training:

‘A registrar took us for one of our lectures … and I thought, “Wow that’s interesting … you completed your medicine.” It was the first time I got to know there was such a specialty’ (FGD3).

However, a theme in all interviews was positive, formative, community exposures, which participants returned to ‘many, many years later’:

‘We went to a rural area. We left our luxury cars, took a bus, went to a clinic. It was the first time we saw the struggles of people, how far they had to travel to come there’ (IDI 1).

### Why specialist training?

#### Practical issues

A few in each focus group started MPHs whilst employed but found it difficult to juggle academic work, classes and full-time jobs. Full-time employment through the specialist/MMed training was an advantage. Despite taking a cut in pay, the combination of broad theoretical PH training and experience, honing skills and exposures for future work, was important:

‘On the MPH you learn a lot of the “What?” and [in] the MMed you get the tools of the “How to” make that change and really get equipped. That appealed to me’ (FGD2).

#### MPH limitations

Registrars felt they differed from physicians doing MPHs or masters in epidemiology, who were developing skills in research or management for *existing* careers. Because nationally MPH degrees are not standardised, they were seen as having variable quality. In contrast, the accredited MMed/fellowship was valued:

‘The MMed, everyone is writing under one college, one exam versus the MPH which is [offered by] different universities’ (FGD3).

#### PHM’S added value

**Figure 2.**
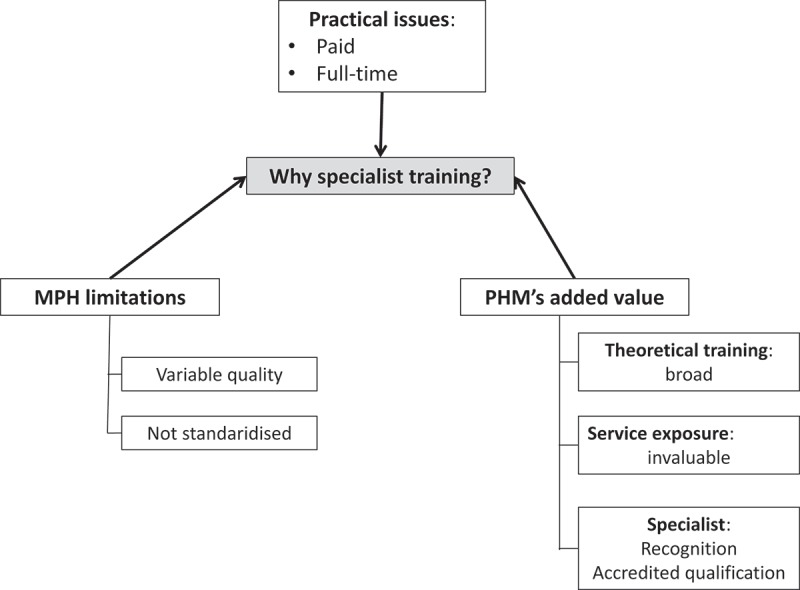
Selecting specialist rather than MPH training.

The value of becoming specialists was raised in all interviews, and elaborated as ‘you’re the “go-to” person’. This differentiated PHM specialists from other PH professionals and in the public sector commanded higher salaries, on par with other medical specialists. Factors underlying informants’ preferences for specialist training are depicted in Figure 2.

### Training – time in the trenches

It was evident from the interviews that both theoretical and practical training were valued. The broad theoretical training, particularly in quantitative research methods and epidemiology, was highlighted:

‘The academic training was excellent, expos[ing] us to high quality methods and evidence-based practice. I still use that all the time’ (IDI 2).

Gaps raised in theoretical training included teaching in monitoring and evaluation, management, health economics, qualitative research methods, using evidence for decision-making, environmental health and scientific writing.

Overwhelmingly, practical training was valued. Registrars appreciated exposure to a range of health institutions, and new specialists remarked that immersion in health services subsequently helped them multi-task. They adapted research skills for project planning and grant-writing, and believed such versatility was uncommon among other PH professionals:

To compare a Public Health Medicine specialist to somebody who’s done just a public health [course] … We can write and talk about most things. We just have a sense of what’s going on in the world. I think we have more to offer. You need time in the trenches, there’s nothing that can replace time and exposure to a very broad curriculum (IDI 3).

New specialists highlighted their exposure to high-level management meetings which enabled them to understand decision-making processes. They recalled being frustrated by service experiences – ‘rotations’ – that were removed from service beneficiaries, and recommended ‘rotations’ closer to service settings, to learn decision-making grounded in community needs. They highlighted that advocacy training, an important part of PH practice, could assist them managing services in underserved communities.

All focus groups highlighted frustrations in some service placements such as filling personnel gaps, holding weak programmes together. A minor theme was the ‘slowness of PH work’. This was experienced as the slow pace of work outputs which physicians found difficult, as they were accustomed to the quick turnaround of clinical work, and the tardiness and resistance to change in adopting innovation in service settings:

What I don’t like is that everything goes on forever. You never finish something up in those six months. The project drags on (FGD3).

In [] province, if you pitched an idea there was a long list of excuses that you had to argue against … ‘We don’t have the budget, we don’t have the staff, it’s not in the [plan], someone else is doing it, we’ve tried that before’ (IDI 2).

A widespread sentiment was that supervising academics were removed from and did not understand service challenges. However, academics with service responsibilities were role models:

‘We don’t get enough mentorship from our profs. We’re left to fend for ourselves. Many times we knew more about the health system, but I do think they can capitalise on their strengths. They have a lot to offer’ (IDI 3).

### Career options

Career paths were seen as uncertain yet emerging. While no positions for PHM specialists are designated in SA’s health system, informants anticipated jobs being created for PHM specialists at all levels of the health system, both managerial and technical (for example, epidemiology), to improve service functioning. The suitability of PHM specialists for senior management posts was raised in all interviews. Participants considered working as district managers or as specialists managing PH ‘units’, providing information/‘intelligence’ required for decision-making, and identifying gaps that could lead to operational research:

I see a technical role where they look at the big picture. They look at the outputs of the services, the data. They advise in terms of interventions and innovations from an evidence base. They should understand how the services work, provide that technical expertise, and do what busy managers just can’t get to do (IDI 3).

However, a dominant theme was that participants believed they would need to market themselves, creating jobs as, in the public sector, politically directed decision-making limited the effectiveness of work. Consequently, they foresaw work in NGOs that supported health service improvement.

[Managers] would say: ‘Who are you, this young boy coming from school, and what is it that you’re going to tell us?’ So it becomes difficult, that resistance. Why not go for an NGO job or any other management-related job? (FGD1).

While no one intended to become an academic immediately, they believed linkages between services and academia as mutually beneficial. Participants saw that employment in both university and service settings was attractive – teaching future health professionals and conducting relevant research.

### The future and profile of PHM

There was optimism for PHM’s future, and informants believed that policymakers recognised the value of PH professionals who could work technically in provincial and district offices, managerially in institutions, as well as in academia:

‘I was so excited! Everyone from the Minister’s office down to the medical school was talking about the changes. It felt like the dawn of public health in South Africa’ (FGD1).

However, they were concerned that PHM specialists would not be selected if job-seekers, including physicians, with MPHs could be appointed at lower salaries. This linked to their perception that the value of specialists was not widely known.

A pervasive theme was the speciality’s invisibility. Whilst this seemed to be changing, participants believed this was due to roles appearing vague to other medical specialists, health professionals, medical students, service managers and the public. They believed the speciality should focus on making itself known:

‘Nobody … understand[s] what Public Health Medicine is … There needs to be good marketing for public health. We need to get out there and show people what … we do’ (IDI 1).

## Discussion

Despite PHM’s uncertain identity and ill-defined career paths, registrars were optimistic about their own and the speciality’s future. They specialised for careers to transform the services that they experienced negatively as junior physicians. Most planned careers that address social determinants of disease, improving communities’ health status. The theme of strengthening health systems is congruent with international PH perspectives [16], and systems-level roles are emphasised in recent approaches to health professional education.

They highlighted that although the speciality was becoming more visible, further advocacy was required as there were few service positions for specialists. Nonetheless, because of the value of their training compared to MPHs, they believed they could find jobs they wanted.

### Motivations

Hirschman’s *Exit, Voice and Loyalty* framework explained participants’ motivations for specialising [15]. Rather than ‘exiting’ the profession, they left frontline clinical medicine and chose roles, driven by ‘loyalty’ to communities, to promote the health of whole populations. Through specialising, they intended becoming change-agents, exercising ‘voice’ to transform services. Stevens usefully applied this framework in her study of SA junior physicians’ service experiences, finding they also felt loyal to communities but had few options to voice this [16].

However, motivations such as the ‘external’ value of becoming a specialist and PHM suiting people with a mathematical bent – an ‘internal’ reason – do not fit neatly in this model. Motivation for prestige and specialist salaries were highlighted by the USA Institute of Medicine for Preventive Medicine specialists [17]. A British PH study also found that motivations for specialisation included satisfying mathematical aptitudes and wishes for predictable working hours [18].

Registrars’ motivations for specialisation – including acquiring skills to improve health on a population level and qualifications to work as planners, implementers and researchers – were also found in a 2014 New Zealand study on PHM physicians’ identity [19]. As the New Zealand study found, this career move was experienced as a potential loss – giving up clinical ambitions and trainees questioning if they would still be physicians.

It is unsurprising that medical training provided little motivation to embark on PH careers, as student physicians are socialised to become clinicians [18,20,21]. However, it is concerning that most trained as physicians within the last 15 years, when SA educational reform adopted bio-psycho-social approaches to health [21]. Participants’ recommendations that PH should be integrated in clinical teaching, complemented by community exposures, research that ‘digs in the determinants of health’ coupled with health promotion, also emerged from other local research [22]. Such approaches need to be researched and evaluated locally.

Their mix of experiential and broad theoretical training was attractive and this distinguished them from physicians with MPHs. SA MPH programmes are largely curriculum-focused with written assignments, exams and a dissertation component. The value of practical training is highlighted in PH education literature [23] and should be considered in other local PH programmes.

Poor congruence between what is needed in PH practice and what is taught is an international phenomenon [11,24]. Important skills-gaps were identified, including monitoring and evaluation, and generating evidence for decision-making. This is core to PH training elsewhere [11], and is valued by PH employers globally [25]. The inadequate attention to management and leadership competencies reported requires attention as these are central to PH physician responsibilities in both high- and middle-income countries such as the USA, Canada, the UK, France, Italy and Japan [11], and Ghana [26]. Recommendations to embed mentors in services, ensuring that teaching reflects skills needed in practice, are echoed in the US as a call for ‘academic health departments’ – partnerships between health agencies and academia [17].

### Career paths, scope of practice and the identity of PHM

The Primary Health Care (PHC) approach and roles for PH-trained physicians are identified in national policies [1,3]. Nonetheless, ill-defined career structures and competition for jobs, concerns echoed in other countries [27], have preoccupied the SA speciality.

Specialists in service posts could be role models, attracting physicians to specialise. PHM specialist posts, recently created in two provincial departments, have been used to advocate for positions in other provinces [28]. Participants were largely optimistic, believing PHM specialists would find niches in this policy environment. In addition, despite there being few specialist positions in SA’s public sector, PH competencies are highlighted locally as core to effective district health management [29].

Possible career paths included health system strengthening at all levels. This could be working in ‘PH units’, as was proposed in the 2011 human resources policy document [3]. Interestingly, in 1994, such units were identified as a resource for ‘critical intelligence’ [30].

Registrars desired district-based work, and, historically, PH-trained Medical Officers of Health worked at local government levels in SA [31], the UK [32] and Canada [11]. In contemporary England, PH specialists are central to local health services [33]. In African countries such as Uganda [4] and Ghana, where the School of Public Health trained most district directors of health [34], local-level PH professionals are also important.

The invisibility of PHM with an ill-defined niche is also noted in recent literature about the speciality in the UK [11], New Zealand [18] and Canada [35], and amongst medical students in France [11]. In the US [16], Ghana [36] and New Zealand [19], PH physicians are comparatively poorly regarded and remunerated. Highlighting these challenges, promoting discussion and research to inform roles in different country contexts, will assist in refining competencies and advocacy for the speciality to bridge the gap between health services and burdens of disease [11].

### Limitations

Although informants came from three institutions, they comprised almost half the cohort nationally and reflected the gender distribution of registrars. Their work experience included a range of contexts and most SA institutions training physicians. All new specialists interviewed were women and therefore the perspectives of men were not elicited. Nonetheless, the study, being qualitative, did not aim to generate findings applicable to all PHM registrars in SA. The researcher (VEMZ), an academic PHM specialist, may have hindered negative responses. A bias may have been introduced by only one transcript being reviewed by a second researcher. Although the research was conducted five years ago, the findings are relevant as career paths for PHM remain unresolved in SA’s restructuring under NHI. Its introduction over the next decade will require high levels of public health skills.

## Conclusion

Despite few positions in the public sector, physicians undergo demanding training to become PHM specialists. Although registrars were partly motivated by ‘push’ factors from negative clinical experiences, they were ‘pulled’ towards PH/PHM, motivated by ‘loyalty’ to ‘voice’ their commitment to people’s health. Their ‘voice’ was to impact on services to improve the health status of SA’s population, and pursued PHM specialist training to launch careers in technical and service functions within district, provincial and national institutions.

The stressful and ‘inhumane’ working conditions of SA junior physicians are widely reported [37], resulting in some ‘exiting’ public sector work [38]. Advocacy is being conducted to address difficult working conditions with policymakers [39]. Further work is required to devise strategies to facilitate ‘voice’ and affirm the ‘loyalty’ that many physicians feel. In the context of PHM, as was argued by participants, this requires creating career paths with earmarked responsibilities for population-oriented PH intelligence, essential for health system transformation.

These findings could be used to attract physicians to PH careers and inform local training to produce appropriately skilled graduates in the numbers needed. Medical curricula that demonstrate the value of PH approaches should attract physicians to PHM careers. The overhaul of SA’s health system, proposed in the *PHC re-engineering policy* [40] and the forthcoming NHI [1] presents an opportunity for the profession to demonstrate its value, providing ‘intelligence’ for effective service functioning and contributing to strengthen the health system.

Further research in different country contexts, and debate – addressing questions of unclear identity and prominence, anticipated competition with other PH professionals, unclear career paths and scope of practice, and content of and heterogeneity in training – would fruitfully contribute to position the speciality. This would promote the speciality’s global role in implementing population approaches to address burdens of disease, focussing on determinants of health towards ‘universal health coverage’ and equity.

## Paper context

In South Africa, physicians enrol for Public Health Medicine (PHM) training despite few public sector job opportunities. This qualitative study, investigating training motivations, found physicians were driven by difficult experiences in poorly resourced clinical settings. Rather than ‘exiting’ the health service, PHM specialist training enacted ‘loyalty’ to population health – to provide ‘public health intelligence’ which facilitates sound policymaking and planning. PHM competencies should be refined and positioned to advocate for roles in health sector reform.
